# Oxidative balance score and periodontitis: nonlinear dose-response in NHANES 2009–2014

**DOI:** 10.1038/s41405-026-00410-7

**Published:** 2026-03-13

**Authors:** Chong Gao, Miaoran Wang, Hairong He, Zhengchuan Zhu, Qiuyan Li, Zhuye Gao, Liqin Mei

**Affiliations:** 1 Department of Pediatric Dentistry, School and Hospital of Stomatology, Wenzhou Medical University, Wenzhou, China; 2 Department of Cardiovascular, Xiyuan Hospital, China Academy of Chinese Medical Sciences, Beijing, China; 3 The First Affiliated Hospital, College of medicine, Xi’an Jiaotong University, Shannxi, China

**Keywords:** Periodontitis, Dental public health

## Abstract

**Background:**

Periodontitis is a chronic inflammatory disease associated with oxidative stress. The Oxidative Balance Score (OBS) is a composite measure of dietary and lifestyle factors that reflect the balance between pro-oxidants and antioxidants. Recently, there has been growing interest in research examining the connection between oxidative balance scores (OBS) and periodontitis. However, the nature of this relationship, particularly whether it follows a linear or non-linear pattern, remains unclear. This study aimed to explore association between OBS and periodontitis using data from the National Health and Nutrition Examination Survey (NHANES 2009–2014.).

**Methods:**

We conducted a cross-sectional study using data from NHANES 2009–2014. A total of 10,714 participants with a mean age of 32.60 years (SD = 24.91) were included in this study. Periodontitis was defined based on clinical periodontal examinations, and OBS was calculated using dietary and lifestyle factors. A generalized additive model was used to explore the nonlinear relationship between OBS and periodontitis. A two-piecewise linear regression model was employed to identify the threshold effect of OBS on periodontitis.

**Results:**

The study found a nonlinear relationship between OBS and periodontitis. When OBS was less than 16, the odds ratio (OR) for periodontitis was 0.74 (95% CI: 0.50–1.09, *P* = 0.13). However, when OBS was greater than 16, the OR for periodontitis significantly decreased to 0.14 (95% CI: 0.09–0.20, *P* < 0.001). The threshold effect of OBS on periodontitis was identified at 16.

**Conclusion:**

This study demonstrates a nonlinear relationship between OBS and periodontitis, with a significant reduction in the risk of periodontitis when OBS exceeds 16.

## Introduction

Periodontitis is a prevalent inflammatory disease affecting the oral cavity, characterized by the destruction of periodontal tissues and subsequent bone loss [1]. It is a significant contributor to tooth loss among adults and has been associated with various systemic conditions, including cardiovascular disease, diabetes, and Alzheimer’s disease [2, 3]. The pathogenesis of periodontitis is closely linked to oxidative stress, which arises from an imbalance between pro-oxidants and antioxidants [4–6]. This oxidative stress is marked by neutrophil infiltration, increased protease secretion, and the production of reactive oxygen species (ROS), all of which contribute to the progressive destruction of periodontal structures and the development of systemic comorbidities such as diabetes and hypertension [3, 7].

The Oxidative Balance Score (OBS) serves as a composite measure that integrates dietary and lifestyle factors to evaluate overall oxidative balance. A higher OBS reflects a greater antioxidant capacity relative to pro-oxidant exposure, which is crucial for mitigating oxidative stress [8]. Recently, there has been growing interest in research examining the connection between oxidative balance scores (OBS) and periodontitis [8, 9]. Studies have shown that OBS is negatively correlated with the occurrence of periodontal disease, suggesting that higher OBS may be associated with a lower risk of periodontal disease. However, the nature of this relationship, particularly whether it follows a linear or non-linear pattern, remains unclear.

To tackle this gap, we conduct dose-response analysis to explore the relationship between OBS and periodontitis utilizing data from the National Health and Nutrition Examination Survey (NHANES) 2009–2014 cycles. We hypothesize that subjects with higher OBS would have lower odds of periodontitis. We aim to explore that OBS has a threshold effect on periodontitis, with a significant reduction in risk beyond a certain OBS level.

## Methods

### Study population

**Fig. 1:**
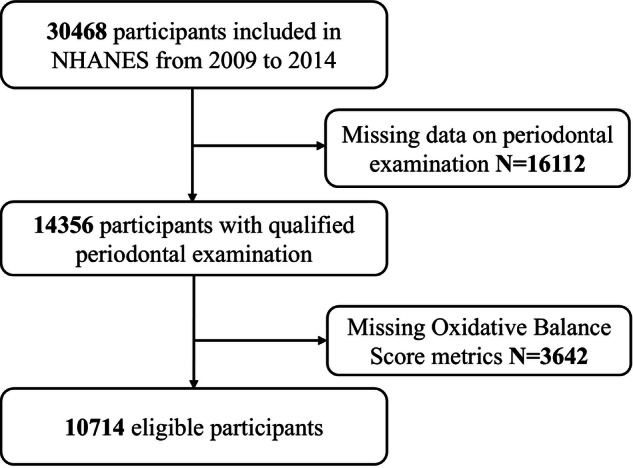
Flow chart of study participant.

The National Health and Nutrition Examination Survey (NHANES) follows the STROBE guidelines [10]. This cross-section study utilized data from the NHANES 2009–2014 cycles, a nationally representative survey conducted by the Centers for Disease Control and Prevention (CDC). NHANES collects health and nutritional data through interviews, physical examinations, and laboratory tests. Overall, 30,468 participants took part in the interview (Fig. 1). A total of 14,356 people from the NHANES had complete periodontal examination records from 2009 to 2014. After excluding 3642 individuals due to missing Oxidative Balance Score information, the cross-sectional study included 10,714 eligible participants.

### Variables

#### Exposure

The Oxidative Balance Score (OBS) was calculated based on 16 dietary factors (carotenoids, dietary fiber, riboflavin, niacin, vitamin B6, total folate, vitamin B12, vitamin C, vitamin E, calcium, magnesium, zinc, copper, selenium, total fat, and iron) and 4 lifestyle factors (physical activity, alcohol consumption, body mass index (BMI), and serum cotinine levels) [11, 12]. Consistent with prior research [13, 14], total fat, iron, alcohol, smoking, and BMI were considered pro-oxidants, while the remaining components were classified as antioxidants.

Dietary data were collected via NHANES interviews. Alcohol intake was assessed as the average number of drinks per day over the past year, and smoking status was evaluated using urinary cotinine levels. BMI was calculated as weight in kilograms divided by height in meters squared. Physical activity was measured using a validated questionnaire.

Each factor was divided into tertiles and assigned scores from 0 to 2, with antioxidants scored positively and pro-oxidants inversely. Nutrient OBS and lifestyle OBS were computed separately and then summed to obtain the total OBS. Further details are provided in Supplementary Table S1.

#### Outcome

Periodontitis was defined based on clinical periodontal examinations, including measurements of pocket depth (PD) and clinical attachment loss (AL). Participants were classified as having periodontitis if they met the criteria set by the CDC/American Academy of Periodontology (AAP) [15]. Mild periodontitis was defined as having either two or more interproximal sites with attachment loss (AL) of at least 3 mm and probing depth (PD) of at least 4 mm (not on the same tooth), or one site with a PD of 5 mm or greater [16]. Moderate periodontitis is characterized by having at least two interproximal sites with attachment loss (AL) of 4 mm or more, or at least two interproximal sites with probing depth (PD) of 5 mm or more, provided these sites are not on the same tooth [17]. Severe periodontitis is defined as having at least two interproximal sites with attachment loss (AL) of 6 mm or more, not on the same tooth, and at least one interproximal site with a probing depth (PD) of 5 mm or more. Individuals not classified within these categories were deemed free of periodontal disease. In our study, periodontitis was classified into two categories: yes (encompassing mild, moderate, and severe cases) and no.

#### Covariates

According to a previous study [8, 9], potential confounders included age, sex, race/ethnicity, education, income-to-poverty ratio (PIR). Additionally, the determination of previous diseases (diabetes, hypertension, cancer) was based on whether the questionnaire indicated that a doctor had previously notified the patient of these issues. The NHANES Survey Methods and Analysis Guide provides thorough information on the methods used for collecting variables.

### Statistical analysis

Analyses utilized sampling weights to account for the NHANES survey’s complex design. OBS were categorized according to medians with interquartile ranges, as follows: Q1(0–12), Q2(13–19), Q3(20–24), Q4(25–36). Due to the skewed distribution of OBS in our study, we performed a Log10 transformation for data analysis. Categorical variables are presented as percentages (*n*%), and continuous variables with a normal distribution are shown as means±standard deviations. The chi-square test (categorical variables) and one-way analysis of variance (ANOVA) (continuous variables) were used to calculate differences between two groups (No periodontitis and periodontitis).

A generalized additive model (GAM) was used to explore the nonlinear relationship between OBS and periodontitis. Adjusted Model was adjusted for age, gender, race/ethnicity, education, poverty index, hypertension, cancer, diabetes. Multiple imputation techniques were employed to address missing covariate data.

A two-piecewise linear regression model was employed to identify the threshold effect of OBS on periodontitis. We also performed a log-likelihood ratio test and compared the one-line linear regression model with the two-piece-wise linear model [18]. We used the bootstrap resampling method to calculate the 95% CI for the turning point. To examine the robustness of the results, we conducted sensitivity analyses. All analyses were performed using R software (version 4.3) and EmpowerStats (version 4.1).

### Ethics approval and consent to participate

The study was approved by the NCHS Ethics Review Board, and all participants provided written informed consent.

## Results

### Baseline characteristics

In this study, a total of 10,714 participants were analyzed, the overall mean observation score (OBS) was 19.09 ± 7.92, with a mean age of 32.60 ± 24.91 years (Table 1). The demographic distribution revealed a near-equal gender ratio, with 49.59% (*n* = 5225) identifying as male and 50.41% (*n* = 5312) as female. Ethnicity composition shows the highest representation from Non-Hispanic White (41.95%, *n* = 4420). Regarding educational level, 48.26% (*n* = 3001) fell into variable (<High school). Furthermore, Participants with periodontitis were more likely to be older, male, white and have lower socioeconomic status.

**Table 1:** Characteristics of the study population by periodontitis.

Characteristics	Overall	No periodontitis	Periodontitis	*P*-value
*N*	10714	5219	5495	
OBS	19.09 ± 7.92	20.10 ± 7.84	18.12 ± 7.87	<0.001
OBS quartile				<0.001
Q1(0–12) *n* (%)	2421(22.60%)	997 (19.10%)	1424 (25.92%)	
Q2(13–19) *n* (%)	2843(26.54%)	1271 (24.35%)	1572 (28.61%)	
Q3(20–24) *n* (%)	2353(21.96%)	1172 (22.46%)	1181 (21.50%)	
Q4(25-36) *n* (%)	3096(28.90%)	1779 (34.09%)	1317 (23.97.%)	
age (years)	32.60 ± 24.91	47.28 ± 13.33	56.00 ± 14.03	<0.001
Gender				<0.001
Male *n* (%)	5225 (49.59%)	632 (38.30%)	1242 (59.09%)	
Female *n* (%)	5312 (50.41%)	1018 (61.70%)	860 (40.91%)	
Race/ ethnicity				<0.001
Mexican American *n* (%)	2384 (22.63%)	211 (12.79%)	467 (22.22%)	
Other Hispanic *n* (%)	1133 (10.75%)	192 (11.64%)	211 (10.04%)	
Non-Hispanic White *n* (%)	4420 (41.95%)	916 (55.52%)	877 (41.72%)	
Non-Hispanic Black *n* (%)	1957 (18.57%)	246 (14.91%)	430 (20.46%)	
Other Race *n* (%)	643 (6.10%)	85 (5.15%)	117 (5.57%)	
Education level				<0.001
>High school *n* (%)	1776 (28.56%)	286 (17.33%)	748 (35.59%)	
High school *n* (%)	1426 (22.93%)	329 (19.94%)	3(23.17%)	
<High school *n* (%)	3001 (48.26%)	1032 (62.55%)	861 (40.96%)	
PIR	2.23 ± 1.60	3.01 ± 1.65	2.32 ± 1.56	<0.001
Hypertension				<0.001
yes *n* (%)	2174 (33.63%)	690 (33.99%)	719 (45.11%)	
no *n* (%)	4285 (66.29%)	1340 (66.01%)	871 (54.64%)	
Cancer				0.123
yes *n* (%)	547 (9.48%)	183 (9.01%)	168 (10.54%)	
no *n* (%)	5222 (90.52%)	1847 (90.99%)	1426 (89.46%)	
Diabetes				<0.001
yes *n* (%)	737 (7.54%	190 (9.36%)	287 (18.01%)	
no *n* (%)	8841 (90.50%)	1782 (87.78%)	1245 (78.11%)	

Categorical variables are presented as *n* (weighted percentage): the *P* value was calculated by the weighted chi-square test; Continuous variables as mean ± SD: the *P* value was calculated by the weighted.

One-way analysis of variance (ANOVA). Among the 10,714 patients, the amount of missing values for the covariates were 86 (0.8%) for race, 545 (5.1%) for education level, 646 (6.0%) for PIR, 667(6.2%) for Hypertension, 913 (8.5%) for cancer, 721 (6.7%) for diabetes.

*OBS* Oxidative Balance Score, *PIR* Poverty to income ratio, *SD* standard deviation.

### Association between oxidative balance score and periodontitis

In the analysis of the association between observation score (OBS) and periodontitis, both unadjusted and adjusted models demonstrated a significant trend (Table 2). The overall odds ratio (OR) for OBS was 0.97 (95% CI: 0.96, 0.97) in the unadjusted model and 0.97 (95% CI: 0.97, 0.98) in the adjusted model, indicating a consistent reduction in odds with increasing OBS. For the tertiles of OBS, the first quartile (Q1) served as the reference group. The second quartile (Q2) showed an OR of 0.87 (95% CI: 0.78, 0.97) in the unadjusted model and 0.88 (95% CI: 0.79, 0.98) in the adjusted model, suggesting a decreased likelihood of periodontitis. The third quartile (Q3) had an OR of 0.71 (95% CI: 0.63, 0.79) and 0.74 (95% CI: 0.66, 0.83), respectively, while the fourth quartile (Q4) had an OR of 0.52 (95% CI: 0.47, 0.58) in the unadjusted model and 0.54 (95% CI: 0.49, 0.61) in the adjusted model. These results indicate a strong inverse relationship between higher OBS and periodontitis, with the likelihood of periodontitis decreasing progressively across tertiles.

**Table 2:** Logistic regression analyses of the associations between Oxidative Balance Scores and periodontitis.

	Unadjusted model OR (95%CI)	Adjusted model OR (95%CI)
Overall OBS	0.97 (0.96, 0.97)	0.97 (0.97, 0.98)
Tertiles		
Q1	1.0	1.0
Q2	0.87 (0.78, 0.97)	0.88 (0.79, 0.98)
Q3	0.71 (0.63, 0.79)	0.74 (0.66, 0.83)
Q4	0.52 (0.47, 0.58)	0.54 (0.49, 0.61)

Adjusted for age, gender, race/ethnicity, education, poverty index, hypertension, cancer, diabetes.

*OBS* Oxidative Balance Score, *OR* odd ratio, *CI* confidence interval.

### Identification of nonlinear relationship

**Fig. 2:**
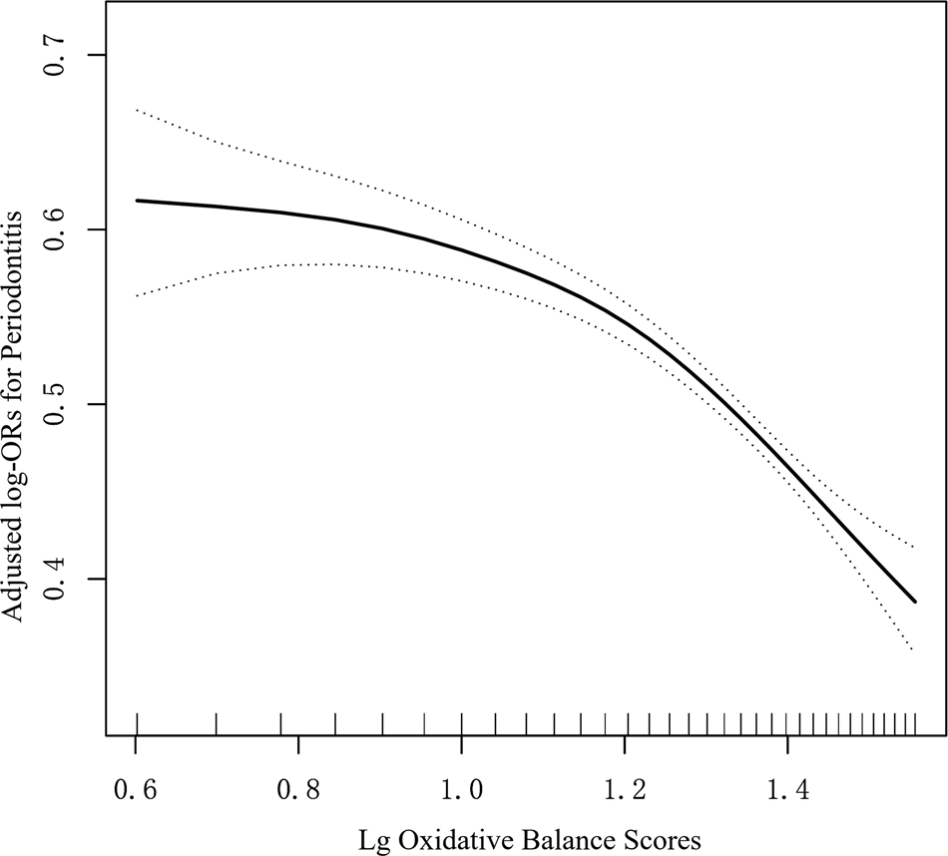
Association between Oxidative Balance Score with periodontitis. Adjusted for age, gender, race/ethnicity, education, poverty index, hypertension, cancer, diabetes. The solid and dotted lines represent the estimated values and their corresponding 95% CIs, respectively.

We observed a nonlinear dose–response relationship between Oxidative Balance Score (OBS) and periodontitis (Fig. 2). The two-piecewise linear regression model confirmed the threshold effect of OBS on periodontitis, and the *P* value of the log-likelihood ratio test was <0.001 (Table 3). The risk of periodontitis decreased significantly as OBS increased, with a threshold effect observed at OBS of 16. Below the threshold of OBS = 16, the association was not statistically significant (OR = 0.74, 95% CI: 0.50–1.09, *P* = 0.13), with a confidence interval spanning the null value. This suggests a weaker and less precisely estimated relationship in the lower OBS range, rather than a clearly flat or null association. Above the threshold, the OR for periodontitis was 0.14 (95% CI: 0.09–0.20, *P* < 0.001).

**Table 3:** Threshold effect analysis of Oxidative Balance Scores in periodontitis patients.

	Adjusted OR (95% CI), *P*-value
Oxidative Balance Scores	
Fitting by the standard linear model	0.32 (0.26,0.39) < 0.0001
Fitting by the two-piecewise linear model	
Inflection point	16
OBS < 16	0.74 (0.50,1.09) 0.13
OBS ≥ 16	0.14 (0.09, 0.37) < 0.0001
P for Log-likelihood ratio	<0.001

Adjusted for age, gender, race/ethnicity, education, poverty index, hypertension, cancer, diabetes.

*OBS* Oxidative Balance Score, *OR* odd ratio, *CI* confidence interval.

In the supplementary analysis, the trend of the sensitivity analysis was consistent with that of the main analysis (Fig. S1). Multiple imputation techniques were employed to address missing covariate data. Similar results were obtained after considering the impact of missing data (Table S2).

## Discussion

This study found a nonlinear relationship between Oxidative Balance Score (OBS) and periodontitis. The key discovery was that the risk of periodontitis significantly decreases when OBS is greater than 16. The strengths of this study include the use of a large, nationally representative sample and the comprehensive assessment of OBS using both dietary and lifestyle factors. To our knowledge, this is the first study to identify and quantify a nonlinear dose‑response relationship with a specific threshold effect (at OBS = 16) in the association between Oxidative Balance Score and periodontitis using NHANES data.

Early studies investigated the association between the Oxidative Balance Score (OBS) and periodontitis in different populations [8, 9]. The first study, by Qu et al., utilized data from the National Health and Nutrition Examination Survey (NHANES) from 1999 to 2018, involving 3706 U.S. adults. The OBS in this study was derived from 16 dietary factors and 4 lifestyle factors, including antioxidants and pro-oxidants. The outcome was periodontitis, classified as mild, moderate, or severe based on clinical attachment loss and pocket depth. The results indicated a negative linear association between OBS and periodontitis, with higher OBS scores associated with a lower risk of periodontitis. Subgroup analyses revealed that this association was more pronounced in older adults and those with diabetes.

The second study, conducted by Seon et al., analyzed data from 9650 Korean adults aged 19 and older, using the 7th Korea National Health and Nutrition Examination Survey (KNHANES) from 2016 to 2018. Oxidative Balance Score, which was calculated based on 5 antioxidants and 5 pro-oxidants. Outcome was the presence of moderate or severe periodontitis, assessed using the Community Periodontal Index (CPI). The study found a negative association between higher OBS and the risk of periodontitis, particularly in men, suggesting that managing oxidative balance may help reduce periodontitis risk.

Similarly, our study corroborates the findings of Seon et al. and Qu et al. regarding the protective role of higher OBS against periodontitis. However, the identification of a nonlinear relationship and a specific OBS threshold in this study adds a novel dimension to the understanding of how oxidative balance influences periodontal health. The differences in findings may be attributed to variations in population characteristics, OBS scoring methods, and the distribution of oxidative stress factors across studies. Future research could explore the mechanisms underlying the threshold effect and investigate whether targeted interventions to increase OBS above this threshold could effectively reduce the risk of periodontitis in diverse populations.

The relationship between the Oxidative Balance Score (OBS) and periodontitis may be mediated through the interplay of oxidative stress and inflammation, both of which are central to the pathogenesis of periodontal disease. A higher OBS, which reflects a greater intake of antioxidants and healthier lifestyle choices, may mitigate oxidative stress by neutralizing reactive oxygen species (ROS) and reducing the production of pro-inflammatory cytokines. Excessive ROS production is a hallmark of periodontitis, leading to tissue damage, DNA oxidation, and lipid peroxidation, which exacerbate periodontal inflammation and bone loss [8, 19]. Oxidative stress is characterized by an imbalance between the production of ROS and the body’s ability to detoxify these harmful compounds or repair the resulting damage. In the context of periodontitis, elevated levels of ROS can initiate and perpetuate inflammatory responses, contributing to the destruction of periodontal tissues [7, 20]. Antioxidants, such as vitamins C and E, along with dietary components like fiber and carotenoids, which contribute to a higher OBS, can enhance the body’s antioxidant defense system. This enhancement helps to reduce oxidative damage and modulate the inflammatory response, thereby potentially protecting against the progression of periodontitis [21, 22]. Moreover, lifestyle factors such as physical activity and smoking cessation, which are integral components of the OBS, further influence systemic inflammation and oxidative balance. Regular physical activity has been shown to improve antioxidant status and reduce systemic inflammation, while smoking is a known risk factor for both oxidative stress and periodontal disease [23, 24]. Thus, a higher OBS may protect against periodontitis by maintaining oxidative homeostasis and attenuating the chronic inflammatory processes that drive periodontal tissue destruction. Research has indicated that specific dietary patterns and antioxidant-rich foods can significantly impact periodontal health. For instance, studies have demonstrated that individuals with higher intakes of antioxidants exhibit lower levels of periodontal disease markers, suggesting that dietary interventions could be beneficial in managing or preventing periodontitis [25, 26]. Furthermore, the role of oxidative stress in the inflammatory response associated with periodontitis underscores the importance of a balanced diet rich in antioxidants as a potential therapeutic strategy [27].

### Study limitations

Our research encountered several limitations. First, one common issue in observational research is the existence of unmeasured confounders. Thus, it is important to carry out large-scale prospective studies in the future to validate the results shown in this study. Second, our study contains incomplete data for certain variables. Nevertheless, we used contemporary methods to deal with missing data to minimize bias. Third, it is noteworthy that the potential resulting from interventions would bias towards to the null and thus result in an underestimation of the association between OBS and periodontitis. Finally, we acknowledge that the participants were patients referred to the U.S adults for some reason, limiting the generalization of the findings to other populations.

## Conclusions

This study demonstrates a nonlinear relationship between OBS and periodontitis, with a significant reduction in the risk of periodontitis when OBS exceeds 16. These findings highlight the importance of maintaining a high OBS through diet and lifestyle modifications to reduce the risk of periodontitis.

## Supplementary information

Supplementary information

STROBE_checklist

## Data Availability

NHANES data are publicly available at http://www.cdc.gov/nchs/nhanes/.
