# Supplementary material for: Oxidative balance score and periodontitis: nonlinear dose-response in NHANES 2009–2014
Source: BDJ Open. 2026 Mar 13;12:21. doi: 10.1038/s41405-026-00410-7 (PMC12988143; doi:10.1038/s41405-026-00410-7)
Supplement: Supplementary file 1 — Supplementary information [file 41405_2026_410_MOESM1_ESM.docx]

| OBS components | Property | Male | | | Female | | |
| --- | --- | --- | --- | --- | --- | --- | --- |
| **Dietary OBS components** |  | 0 | 1 | 2 | 0 | 1 | 2 |
| Dietary fiber (g/d) | A | <13.15 | 13.15-20.7 | ≥20.7 | <11.25 | 11.25-16.95 | ≥16.95 |
| Carotene (RE/d) | A | <667.50 | 667.50-2326.00 | ≥2326.00 | <686.50 | 686.50-2499.00 | ≥2499.00 |
| Riboflavin (mg/d) | A | <1.74 | 1.74-2.51 | ≥2.51 | <1.38 | 1.38-1.96 | ≥1.96 |
| Niacin (mg/d) | A | <22.28 | 22.28-31.66 | ≥31.66 | <16.15 | 16.15-22.87 | ≥22.87 |
| Vitamin B6 (mg/d) | A | <1.73 | 1.73-2.53 | ≥2.53 | <1.30 | 1.30-1.88 | ≥1.88 |
| Total folate (mcg/d) | A | <328.50 | 328.50-490.00 | ≥490.00 | <261.00 | 261.00-383.00 | ≥383.00 |
| Vitamin B12 (mcg/d) | A | <3.64 | 3.64-6.14 | ≥6.14 | <2.65 | 2.65-4.53 | ≥4.53 |
| Vitamin C (mg/d) | A | <42.65 | 42.65-101.25 | ≥101.25 | <40.4 | 40.4-90.45 | ≥90.45 |
| Vitamin E (ATE) (mg/d) | A | <5.88 | 5.88-9.14 | ≥9.14 | <4.78 | 4.78-7.6 | ≥7.6 |
| Calcium (mg/d) | A | <736.00 | 736.00-1110.00 | ≥1110.00 | <618.00 | 618.00-920.00 | ≥920.00 |
| Magnesium (mg/d) | A | <255.50 | 255.50-354.50 | ≥354.50 | <206.00 | 206.00-283.50 | ≥283.50 |
| Zinc (mg/d) | A | <9.71 | 9.71-14.06 | ≥14.06 | <7.23 | 7.23-10.29 | ≥10.29 |
| Copper (mg/d) | A | <1.05 | 1.05-1.47 | ≥1.47 | <0.86 | 0.86-1.20 | ≥1.20 |
| Selenium (mcg/d) | A | <101.00 | 101.00-140.60 | ≥140.60 | <73.45 | 73.45-103.85 | ≥103.85 |
| Total fat (g/d) | P | ≥96.89 | 66.64-96.89 | <66.64 | ≥73.13 | 49.65-73.13 | <49.65 |
| Iron (mg/d) | P | ≥18.17 | 12.58-18.17 | <12.58 | ≥14.02 | 9.81-14.02 | <9.81 |
| **Lifestyle OBS components** |  |  |  |  |  |  |  |
| Physical activity (METminute/week) | A | <620.00 | 620.00-3600.00 | ≥3600.00 | <180.00 | 180.00-1640.00 | ≥1640.00 |
| Alcohol (g/d) | P | ≥30.00 | 0-30.00 | None | ≥15.00 | 0.00-15.00 | None |
| Body mass index (kg/m^2^) | P | ≥30.20 | 25.91-30.20 | <25.91 | ≥31.51 | 25.44-31.51 | <25.44 |
| Cotinine (ng/mL) | P | ≥0.68 | 0.02-0.68 | <0.02 | <0.02 | 0.02-0.10 | ≥0.10 |

**Supplementary Table S1** Oxidative balance score assignment scheme.

A stood for the antioxidant, P for the pro-oxidant, RE for the retinal equivalent, ATE for the alpha-tocopherol equivalent, and MET for the metabolic equivalent.

**Figure S1** Forest plots of stratified analysis of Oxidative Balance Score with periodontitis.
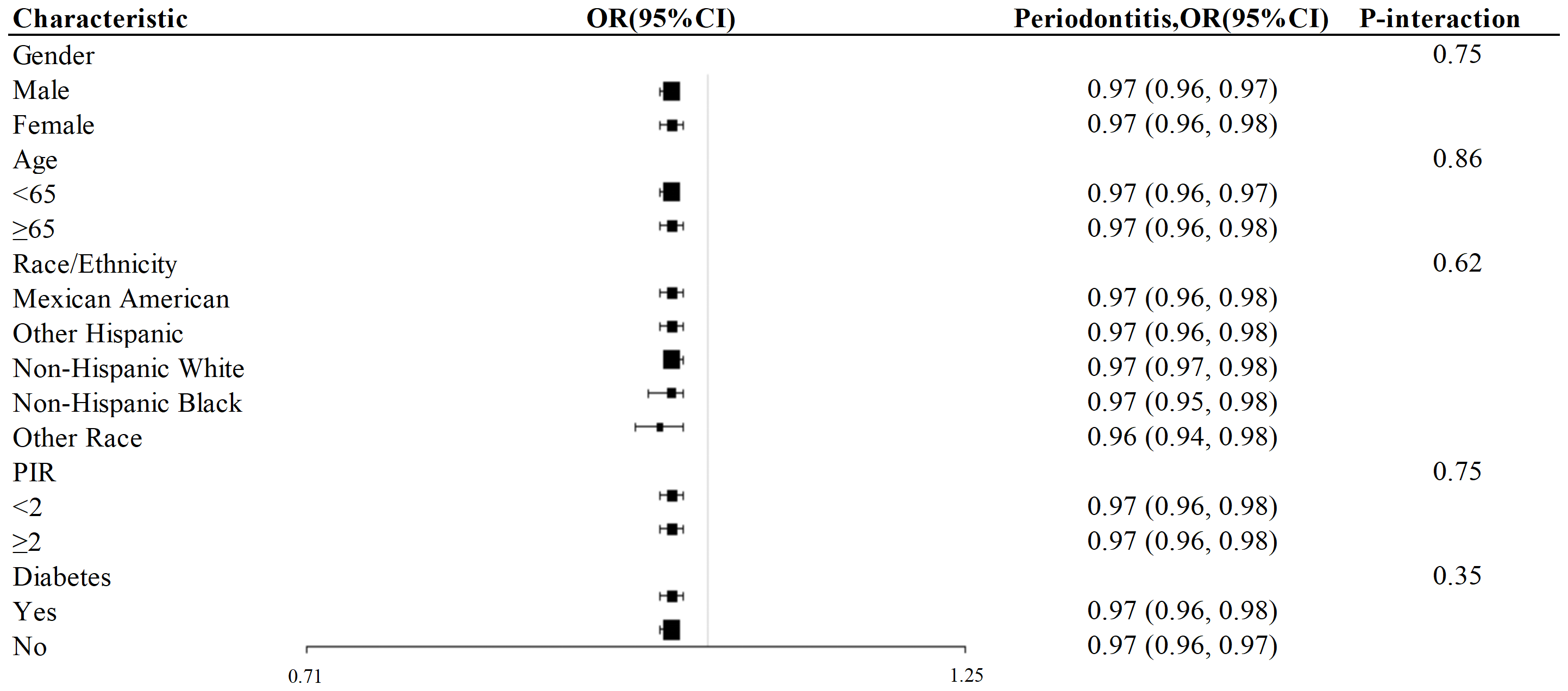


Note: Age, gender, race/ethnicity, education, poverty index, hypertension, cancer, diabetes were all adjusted except the variable itself.

**Table S2** Logistic regression analyses of the associations between Oxidative Balance Scores and periodontitis

|  | Non-adjusted | Adjust I (Complete case) | Adjust II (Multiple imputation) |
| --- | --- | --- | --- |
|  | 0.97 (0.96, 0.97) <0.0001 | 0.97 (0.97, 0.98) <0.0001 | 0.97 (0.97, 0.98)  <0.0001 |
| OBS quartile |  |  |  |
| Q1 | 1.0 | 1.0 | 1.0 |
| Q2 | 0.87 (0.78, 0.97) 0.0101 | 0.88 (0.78, 0.98)  0.0214 | 0.88 (0.79, 0.98)  0.0226 |
| Q3 | 0.71 (0.63, 0.79) <0.0001 | 0.73 (0.65, 0.82) <0.0001 | 0.74 (0.66, 0.83)  <0.0001 |
| Q4 | 0.52 (0.47, 0.58) <0.0001 | 0.54 (0.48, 0.60) <0.0001 | 0.54 (0.49, 0.61)  <0.0001 |

Note: Adjusted for age, gender, race/ethnicity, education, poverty index, hypertension, cancer, diabetes.

Abbreviations: OBS: Oxidative Balance Score; OR: odd ratio; CI: confidence interval
